# 3D-printing techniques in a medical setting: a systematic literature review

**DOI:** 10.1186/s12938-016-0236-4

**Published:** 2016-10-21

**Authors:** Philip Tack, Jan Victor, Paul Gemmel, Lieven Annemans

**Affiliations:** 1 Department of Public Health, Ghent University, De Pintelaan 185, 9000 Ghent, Belgium; 2 Ghent University Hospital, Ghent University, De Pintelaan 185, 9000 Ghent, Belgium; 3 Departement of Economics & Business Administration, Ghent University, Tweekerkenstraat 2, 9000 Ghent, Belgium

**Keywords:** 3D printing, Additive manufacturing, Innovation, Surgery, Review, Patient specific, Customized, Anatomic model

## Abstract

**Background:**

Three-dimensional (3D) printing has numerous applications and has gained much interest in the medical world. The constantly improving quality of 3D-printing applications has contributed to their increased use on patients. This paper summarizes the literature on surgical 3D-printing applications used on patients, with a focus on reported clinical and economic outcomes.

**Methods:**

Three major literature databases were screened for case series (more than three cases described in the same study) and trials of surgical applications of 3D printing in humans.

**Results:**

227 surgical papers were analyzed and summarized using an evidence table. The papers described the use of 3D printing for surgical guides, anatomical models, and custom implants. 3D printing is used in multiple surgical domains, such as orthopedics, maxillofacial surgery, cranial surgery, and spinal surgery. In general, the advantages of 3D-printed parts are said to include reduced surgical time, improved medical outcome, and decreased radiation exposure. The costs of printing and additional scans generally increase the overall cost of the procedure.

**Conclusion:**

3D printing is well integrated in surgical practice and research. Applications vary from anatomical models mainly intended for surgical planning to surgical guides and implants. Our research suggests that there are several advantages to 3D-printed applications, but that further research is needed to determine whether the increased intervention costs can be balanced with the observable advantages of this new technology. There is a need for a formal cost–effectiveness analysis.

**Electronic supplementary material:**

The online version of this article (doi:10.1186/s12938-016-0236-4) contains supplementary material, which is available to authorized users.

## Background

3D printing has become more important in recent decades. 3D printing allows three-dimensional renderings to be realized as physical objects with the use of a printer. It has revolutionized prototyping and found applications in many nonmedical fields. In medicine, the technology has applications in orthopedics, spinal surgery, maxillofacial surgery, neurosurgery, and cardiac surgery, amongst various other disciplines.

Doctors mostly work with two-dimensional X-ray images or two-dimensional images obtained from computed tomography (CT) or magnetic resonance (MR) scans to gain insight into pathologies. This requires excellent visualization skills from the surgeon. The recent emergence of three-dimensional renderings of CT, MR, plain radiography, and echo imagery has improved visualization of complex pathologies but lacks tactile qualities. 3D-printed objects can be used to study complex cases, to practice procedures, and to teach students and patients. [1].

Furthermore, some current surgical procedures are complex and require guidance to avoid damaging essential parts of the body, or to obtain an acceptable esthetic outcome [2]. In some cases, this guidance requires substantial amounts of ionizing radiation and can heavily increase surgical time [3]. Additionally, anatomical defects can require custom prosthetics to repair damage as accurately as possible [4].

The need for improved visualization and surgical outcomes has given rise to 3D-printed anatomical models, patient-specific guides, and 3D-printed prosthetics. The growing surgical applications of 3D printing have made it interesting to analyze the current implementation of this new technology.

This article gives an overview of the current usage of 3D-printing techniques in human medicine, more specifically surgery, based on a systematic literature review using three major literature databases.

We attempted to identify domains and usages where the technology is fairly common or has been used several times, and to report its potential advantages and disadvantages. As healthcare budgets are under pressure and both hospitals and doctors desire to improve efficiency, we have included cost and cost effectiveness as variables in the analysis.

This resulted in the following research questions: (1) which surgical 3D-printing applications are commonly reported in human medicine? (2) What advantages, disadvantages, and cost consequences do surgical 3D-printing applications have compared to the standard of care?

## Methods

A systematic literature review was conducted using the Web of Science, PubMed, and Embase.

The search strategy was kept broad to ensure no relevant papers were excluded. The search headings were ‘3D printing’, ‘three dimensional printing’, ‘additive manufacturing’, and ‘rapid prototyping’. After expert consultation, an additional search was performed to include 3D-printing applications referred to as ‘patient specific’ guides and implants. Relevant articles found in references were added as well.

The initial database search was conducted in February 2015. An additional search was conducted in December 2015, to include all papers published in 2015. Only full papers of controlled trials and case series of minimum four cases, written in English, where 3D printing is applied for surgical purposes on living humans, were considered.

Manual screening of the titles and abstracts was performed so as to include only papers consistent with the application of 3D-printing techniques to human medical ends. The inclusion criteria were the use of ‘computer aided manufacturing’ (CAM), ‘computer aided design’ (CAD), ‘additive manufacturing’ (AM), ‘printed scaffold’,’stereolithography’, and ‘reverse engineering’ for human medicine. Additionally, titles containing ‘customized’, ‘patient specific’, ‘templates’ and ‘physical model’ were retained in order not to overlook potential uses.

Examples of virtual 3D modeling or rendering without physical 3D models were excluded. Only clinical uses were considered; cadaveric, in vitro, and animal studies were not retained.

Only case series with more than three cases and clinical trials were selected, because we associate these with higher integration of the technology in the medical field. Publications written in languages other than English, or with no full paper available, were excluded based on the abstract.

Papers retained after the full-text review were analyzed in detail using an evidence table to report relevant study characteristics and outcomes. Based on commonly reported outcomes in the literature, we included the following variables: impact on operation room (OR) time or treatment time, level of accuracy of the printed part, impact on exposure to radiation, clinical outcome, cost, and cost effectiveness.

The impact on OR time/treatment time refers to time savings in the operation room or for the treatment itself, compared to the conventional procedure. This does not include savings in rehabilitation, nor does it take account of any additional work done by the surgeon prior to surgery.

The accuracy of the printed part was used to assess the quality of the printed part. For anatomical models, the resemblance to the original form was taken into account. For guides and implants, the accuracy of the printed part was assessed based on intraoperative adaptations and the need to abort the intended procedure in favor of the conventional procedure. The occurrence of few changes to the guide or few procedures being converted to the conventional procedure was considered to reflect good accuracy.

Radiation exposure was captured when mentioned explicitly by authors. Clinical outcome was assessed as improved surgical precision or improved final outcome. Note that there is an overlap between accuracy of the printed part and clinical outcome, as accurate guides result in better postsurgical alignment and therefore a positive outcome score. Cost was captured when mentioned by the authors. As some authors have begun to debate cost effectiveness, we considered this variable when it was mentioned.

## Results

After the initial database search in February 2015, 7482 papers were selected. The additional search in December 2015, including all 2015 publications, resulted in 1114 papers. 3386 duplicates were removed. Screening of titles resulted in 1873 retained articles, with 2223 articles being excluded.

353 papers were selected for full reading; 1520 articles were excluded, most of which were case studies.

After reading the full papers, 224 papers were retained for further analysis. With the exception of three papers, all were surgical. Nonsurgical papers were excluded. Six relevant papers found in references of the accepted papers were added to the final analysis table, bringing the total number of papers to 227.

An overview of the selected papers ranked by medical domain is given in Additional file 1. One paper was split in three, as three different studies were published together. Another paper was split in two since two different studies were discussed in it. This resulted in 230 observations in the 227 included papers.

**Fig. 1:**
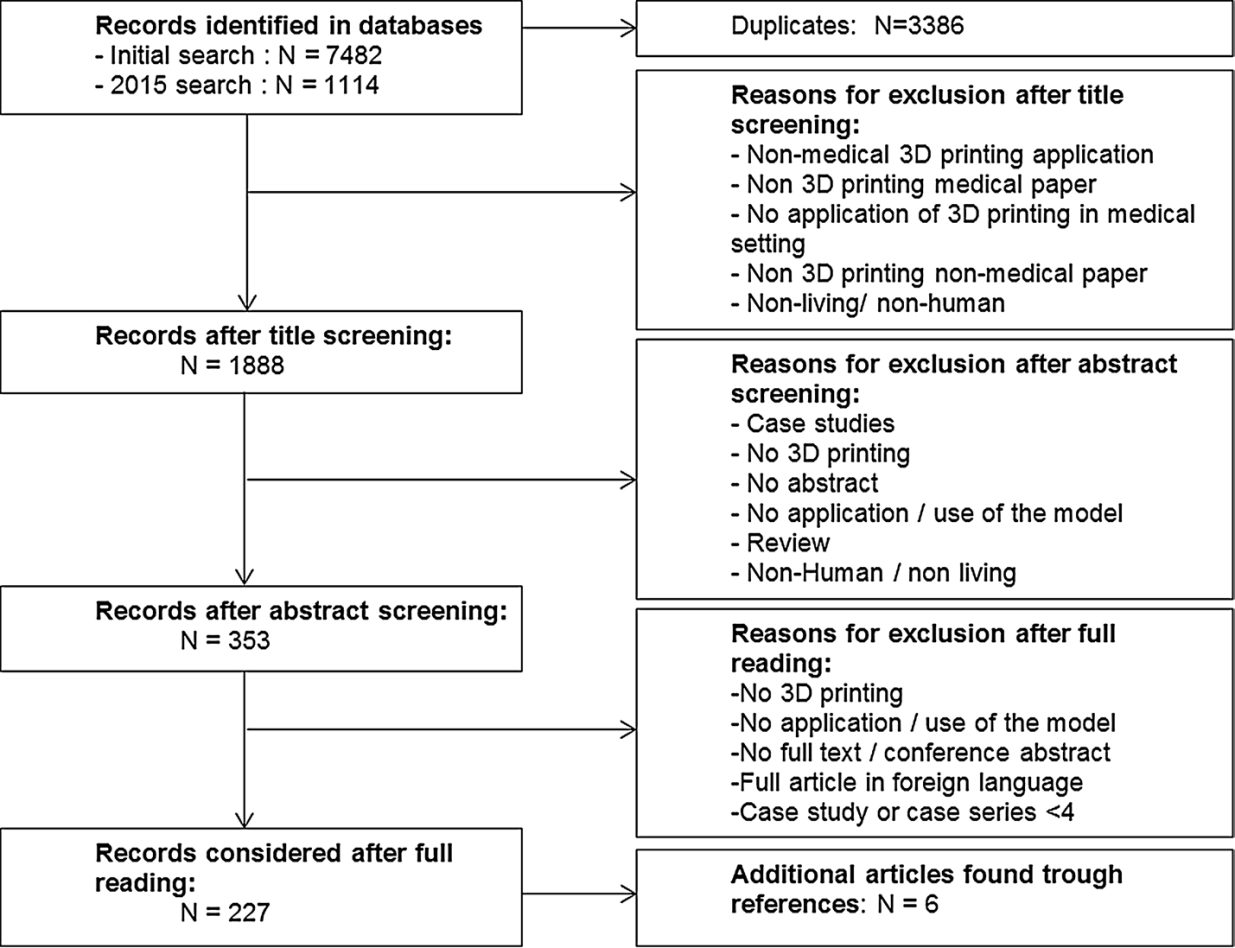
Search strategy and reasons for exclusion

The search strategy and reasons for exclusion are given in Fig. 1.

**Fig. 2:**
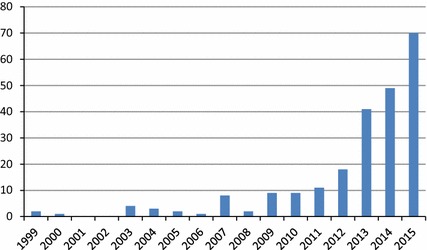
Overview of selected papers based on publication year

Only two papers were dated before 2000. Eight papers were dated between 2000 and 2005, 30 between 2006 and 2010, and 189 between January 2011 and 25 February 2015. Figure 2 gives an overview of the number of selected papers per year.

**Fig. 3:**
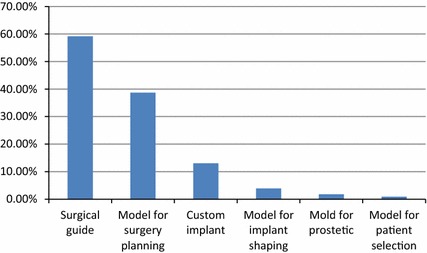
Overview of the usage of 3D-printing techniques as percentage of total number of papers

The published results on 3D printing most often concern surgical guides (60 %) and models for surgical planning (38.70 %) (Fig. 3). Additionally, there are reports on the outcomes of using 3D printing to make custom implants (12.17 %), molds for prosthetics (3.91 %), models of implant shaping (1.74 %), and models for patient selection (0.87 %). Note that some papers used 3D-printing techniques for multiple purposes, resulting in a total greater than 100 %.

**Fig. 4:**
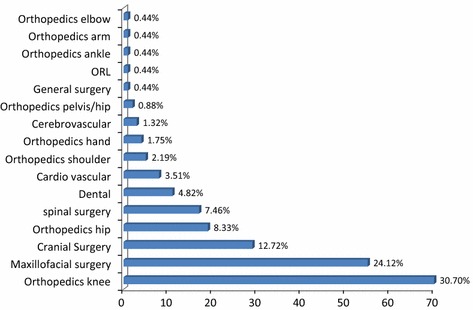
Overview of papers per specific field

The reports on 3D printing outcomes concern multiple surgical domains. Orthopedics has the largest share, with 45.18 % (Fig. 4): this is made up of knee (30.70 %), hip (8.33 %), shoulder (2.19 %), and hand (1.75 %) orthopedics. Maxillofacial surgery also accounts for a large share (24.12 %). This is followed by cranial surgery and spinal surgery, representing 12.72 and 7.46 % respectively.

More in-depth results are collected in an overview table (Table 1). The data is organized by usage of the technology and discipline. An overview of the number of papers is given in each category. The total of 270 exceeds the total number of papers, as one paper can address multiple usages of 3D printing. The first variable in the table is impact on operation room (OR) time/treatment time. Reductions in operating time are assessed as beneficial. Secondly, the accuracy of the printed part is evaluated. As explained above, radiation exposure is only taken into account when the change in radiation exposure is explicitly mentioned in the paper. Medical outcome and cost are the final regular variables. The last of these, cost effectiveness, is only reported when the authors explicitly mention cost effectiveness. A broader version of the evidence table can be found in Additional file 2.

**Table 1:** Evidence table

Number of studies	Custom implant	Model for implant shaping	Model for patient selection	Model for surgery planning	Mold for prosthetic	Surgical guides	Total
	30	9	2	89	4	136	270
*OR/treatment time*							
Not mentioned	11	4	2	37	3	68	125
Time reduction	17 (4)	5 (1)	0	48 (13)	1	53 (28)	123 (46)
No time difference	1 (1)	0	0	3 (2)	0	8 (1)	12 (4)
Time increase	1	0	0	2 (1)	0	7 (5)	10 (6)
*Accuracy of printed part*							
Not mentioned	3	1	1	4	0	16	28
Good/better accuracy	26	8	1	80 (4)	4	87 (13)	205 (17)
Average accuracy	1	0	0	6 (1)	0	23 (3)	30 (4)
Bad accuracy	0	0	0	0	0	10 (6)	10 (6)
*Radiation exposure*							
Not mentioned	30	7	2	77	4	121	241
Less radiation	0	0	0	8 (1)	0	9	17 (1)
equal radiation	0	0	0	1	0	2	3
Increased radiation	0	2	0	3	0	4	9
*Clinical outcome*							
Not mentioned	1	0	2	10	0	15	28
Improved	25 (2)	9 (2)	0	73 (8)	4	85 (15)	195 (27)
Equal	4	0	0	7 (1)	0	30 (7)	41 (8)
Negative impact	0	0	0	0	0	7 (2)	7 (2)
*Cost*							
Not mentioned	16	7	1	52	3	94	173
Cheaper	0	0	0	4	1	2 (1)	7 (1)
Equally expensive	0	0	0	1	0	1	2
More expensive	14 (4)	2 (2)	1	32 (21)	0	39 (19)	88 (46)
*Cost effectiveness*							
Cost-effective	1	0	0	8	1	10	19
Neutral	0	0	0	2	0	1	3
Not cost-effective	0	0	0	1	0	6	7

*(x)* Number of studies quantifying the data with numbers/statistics

### Custom implants

Custom implants are used in cranial surgery, dentistry, and maxillofacial surgery [4–32]. According to 17 out of 28 papers, custom implants reduce OR/treatment time. 25 papers mentioned good accuracy of the custom implants and improved medical outcomes. Radiation exposure was not mentioned in these papers. 14 papers mentioned increased costs, but one described an increase in cost effectiveness [4].

The custom implants were mostly made of titanium (10 of 28), polyether ether ketone (PEEK) (10 of 28), epoxide acrylate hydroxyapatite (2 of 28), hydroxyapatite (2 of 28), polymethyl methacrylate (1 of 28), polypropylene–polyester (1 of 28), and nonspecified acrylic-based resin (4 of 28).

### Anatomical models

Anatomical models can be used for implant shaping in maxillofacial surgery, a topic that was discussed in nine studies [33–41]. Five papers mentioned time reduction as advantage [33, 36, 38–40]. Eight studies concluded that printed models provide good anatomical representations and nine studies mentioned improved surgical outcomes. Two studies mentioned exposure to ionizing radiation [36, 41] and two mentioned increased costs [39, 41].

Anatomical models are also used in selecting patients for cardiovascular surgery; this was discussed in two studies [42, 43]. None of the papers mentioned time reductions, exposure to ionizing radiation, or medical outcome. One paper found the model to be a good representation of the actual pathology but did not mention the associated costs [42]. Another publication mentioned that costs increased as a result of using an anatomical model [43].

Multiple domains use anatomical models for surgical planning. Our research showed anatomical models being used in cardiovascular surgery, vascular neurosurgery, dental surgery, general surgery, maxillofacial surgery, neurosurgery, cranial/orbital surgery, orthopedics, and spinal surgery [1–3, 9, 14, 15, 35, 37, 39, 43–121]. Among the 89 studies, 48 (53.93 %) mentioned reduced operation room time. Two (2.24 %) studies mentioned increased operation room time and 37 (41.57 %) did not mention any impact on operation room time. Only 13 of the 48 studies mentioning reduced operation room time and supported this statement with actual numbers or statistics [3, 39, 44, 72, 74, 78, 81, 84, 99, 107, 117, 119, 120]. In 80 (89.89 %) of the publications, the printed part showed good accuracy, although this was only supported numerically in four studies [3, 81, 97, 106]. Exposure to ionizing radiation was not mentioned in 77 (86.51 %) of the publications, and eight mentioned decreased exposures [3, 59–61, 74, 79, 101, 107]. Three publications mentioned increased exposure to ionizing radiation [92, 111, 114]. No publication mentioned decreased medical outcomes with the use of anatomical models, while 73 publications mentioned improved medical outcomes. On the cost side, 52 publications did not mention costs, four mentioned decreased costs, and 32 mentioned increased costs. Two-thirds of the studies reporting increased costs supported this claim with numbers or statistics. Eight studies, of which four used the models for maxillofacial surgery, estimated the anatomical models to be cost-effective [44, 58, 67, 74, 79–81, 97].

### Molds for prosthetics

3D-printing techniques can be used to produce molds for making prosthetics, as discussed in three studies [45, 122, 123]. We encountered this approach in cranial surgery, maxillofacial surgery, and ear surgery. In all the studies, the printed parts were accurate and improved the medical outcome. Both cranial studies were discussed in a single paper. One of these studies mentioned reduced OR time as an advantage [45]. The study using 3D-printed molds for ear prosthetics stated that their use reduced costs and was cost-effective [123]. None of these studies mentioned exposure to ionizing radiation.

### Surgical guides

Surgical guides are the most popular medical application of 3D printing, with mentions in 137 of the 270 papers (50.74 %) [10, 15, 30, 31, 39, 48, 59, 60, 62, 70, 71, 73, 74, 76, 77, 79–81, 83, 84, 86, 88, 89, 92, 93, 96–98, 106, 108, 111–113, 118, 124–226]. Apart from orthopedics (guides for knee arthroplasties), 3D-printed surgical guides were also used in neurosurgery, dental surgery, spinal surgery, and maxillofacial surgery. 28 of the 53 studies that mentioned reduced operation room time also supported this claim with numbers or statistics [39, 74, 81, 84, 118, 131, 132, 135, 136, 140, 141, 145, 151, 152, 162, 175, 177, 181, 190, 194, 196, 200, 207, 210–212, 219]. Increased procedural time was seen in seven papers, of which five supported this with numbers or statistics [62, 73, 125, 143, 153, 161, 225]. 88 studies reported that the guides had good accuracy, while 23 reported average accuracy, and ten mentioned insufficient accuracy. Interestingly, six out of the ten papers reporting insufficient accuracy backed this up with numbers or statistics [148, 165, 182, 185, 191, 211]. Radiation exposure was not mentioned in 123 (89.13 %) studies. Less radiation was mentioned in nine studies, including by six of the 11 spinal surgery studies. Surgical guides improved clinical outcomes in 86 (62.31 %) cases, gave similar results in 31 cases, and had a negative impact on clinical outcome in seven studies, all of which were knee orthopedics. The cost associated with the guides was only mentioned in 42 studies, of which 39 stated it to be more expensive and two stated it to be equally expensive. 19 of the 39 studies which indicated that the new technology was more expensive supported this finding with numbers or statistics. Ten studies stated that the guides were cost-effective, while six stated that they were not cost effective. None of these studies backed these claims with numbers.

Considering all applications, the new 3D-printing technology reduced operation room time in 46 % of the studies. 76 % of the studies mentioned that the printed part had good accuracy, and 72 % mentioned improved medical outcomes. On the other hand, 33 % of authors stated that the technology was more expensive.

### Reductions in operation room time

Operation room time has always been one of the major arguments for medical 3D printing. Of the 227 articles, 42 described the precise impact of using 3D printing technology on OR time. For the majority of applications, 3D printing resulted in time savings. The results are given in Table 2. 3D applications such as surgical guides for maxillofacial surgery, models for spinal and maxillofacial surgical planning, and models for shaping implants used in maxillofacial surgery seem to benefit the most from the technology.

**Table 2:** Reported impact of medical 3D printing on operation room time

		Count	Average (in min)	Standard deviation
Cranial surgery	Custom implant	4	−69.16	92.62
*Cranial surgery*	*Custom implant*	*3*	−*15.81*	*7.74*
Maxillofacial surgery	Model for implant shaping	1	−42	
Cerebrovascular	Model for surgery planning	1	−30	
Maxillofacial surgery	Model for surgery planning	5	−5.8	78.52
*Maxillofacial surgery*	*Model for surgery planning*	*4*	−*43.5*	*24.52*
Orthopedics hip	Model for surgery planning	2	0.75	6.75
Spinal surgery	Model for surgery planning	2	−45.5	17.5
Maxillofacial surgery	Surgical guide	6	−60.33	61.85
Orthopedics ankle	Surgical guide	1	−12	
Orthopedics hip	Surgical guide	4	−0.025	5.72
Orthopedics knee	Surgical guide	20	−6.73	13.68

*Italic text* outlier correction (outlier defined as study with a highly different outcome compared to the average of the remaining studies within the group)

## Discussion

At the time this review was begun, no other analysis of the integration of medical 3D-printing techniques, domain, and use existed. Around mid-2015, Hammad et al. reviewed 93 articles concerning current surgical applications [227]. Both their review and the present one come to similar conclusions. This review is more elaborate, including as it does 227 surgical papers and using a standardized form to evaluate these papers.

One of the main inclusion criteria was the use of 3D-printed materials for in vivo medical purposes. Papers describing 3D models used for medical teaching and testing purposes were therefore not included. Case series of four or more trials were considered, as we believe these reflect the maturity of the technological application for the specific domain. The number of publications meeting our selection criteria is increasing: only two studies were selected from 1999, while there were 70 qualifying studies in 2015, showing the growing interest of the medical sector in 3D-printing technologies. 3D-printed parts have several purposes in the medical setting. While anatomical models made up the biggest share in the early years of medical 3D printing, the growing importance of 3D-printed guides is noticeable. Surgical guides are now the most commonly reported type of 3D-printed application, with 60 % of studies mentioning the use of printed surgical guides.

### Anatomical models

3D-printed anatomical models see broad use in the surgical field. Our review suggests that, in orthopedics, their use has been shown to be beneficial, especially in complex hip replacements, where improved medical outcomes were reported unanimously. Also, studies of cranial (mostly orbital) fractures have reported improved results which have been credited to the use of anatomical models as guides prior to and during surgery, in order to understand the pathology better and to avoid pitfalls. These cranial anatomical models are often also used to shape the implant prior to surgery, resulting in an improved fit of the implant, improved medical outcome, and reduced surgical time. As with the anatomical models used for orthopedic and cranial purposes, our research suggests that spinal and maxillofacial models improve operation planning and clinical outcome, while reducing operation time. Furthermore, anatomical models can reduce the need for fluoroscopy during spinal surgery, reducing exposure to ionizing radiation.

Our research found anatomical models useful for planning vascular procedures such as percutaneous valve implantation, repair of aorta and cranial aneurisms, and surgical planning of complex congenital heart malformations. Furthermore, two cardiovascular studies suggested that the models improve patient selection for endovascular procedures, as compared with standard medical imaging.

Anatomical models can have direct usage during surgical procedures. During tooth transplant surgery, 3D models of teeth are used to prepare the donor site, improving the procedure’s success rates. Furthermore, anatomical models of the mouth are used to make drilling guides for dental implants and to make custom obturators for patients following maxillectomy. The latter reduced the amount of labor-intensive work on the part of both dentists and technicians. Furthermore, maxillofacial models are frequently used to shape implants prior to surgery, further enhancing surgical speed while improving clinical and esthetic outcomes.

Although anatomical models can be used on their own, our study perceived a tendency toward using anatomical models in combination with printed surgical guides. Apart from the previously mentioned benefits, anatomical models can be used for teaching medical students and can improve patient communication and knowledge of the pathology.

### Surgical guides

Our research suggests that surgical guides are well incorporated in orthopedic surgery, spinal surgery, maxillofacial surgery, and dental surgery with more than half of the selected studies of our review mentioning the use of guides. Knee surgeons seem to be most interested in using guides. The uniquely positive results of knee orthopedic papers from 2012 gave way to more neutral results the years after, suggesting the initial excitement was tempered when the technology became more common. More recent studies mention no substantial difference in clinical outcome between patient-specific guides and standard instrumentation for total knee arthroplasty. Increased procedural complexity and less-experienced low-volume surgeons favor the use of surgical guides. Apart from clinical results, patient-specific guides reduce the number of surgical trays needed and slightly reduce OR time. Greater reductions in OR time were when surgeons have become more used to the guided procedure, according to one of the selected papers. Cost-effectiveness remains to be proven, but recent studies mentioning the cost-effectiveness of knee-guides suggest that the technology does not offer enough advantages to cover the additional costs associated with the guides.

Based on our findings, surgical guides seem to reduce operation room time and improve medical outcomes for spinal and cranial surgery. This is due to the simulation on models and the accurate translation of the preliminary surgery by means of guides. More than half of the selected studies reported reduced exposure to ionizing radiation (Additional file 1) due to the decreased need for fluoroscopy. In maxillofacial surgery, 3D-printed models and surgical guides are increasingly used for mandibular reconstructions and orthognathic surgery. The guides are used for the resection of both the mandibular part and the graft, as well as to reconstruct the missing part during oncological mandibular resections and reconstructions. According to the results of our research, spinal surgical guides translate the surgical planning accurately and make the outcomes less dependent on the surgeon’s experience. Similar results are seen with the use of guides during dental surgeries. Some authors question the systematic use of dental guides because of the associated higher costs, and suggest that guides be used only in complex cases. Finally, 3D-printed stereotactic fixtures can be used to guide implantation of deep brain stimulation implants with a substantial reduction of surgical time.

The accuracy of the guide or model and the accurate placement of the guide play important roles in the final clinical outcome or advantage provided by the model. The overlap between accuracy and clinical outcome is therefore unavoidable. The accuracy of guides can vary depending on the manufacturer providing the 3D-printed element and the time between the scan used for the production of the guide and the moment of surgery. Furthermore, surgical experience is needed to detect defective guides. Finally, the use of MRI or CT has an impact on the accuracy of the guide.

### Custom implants

Anatomical models can be used as molds to manufacture prosthetics, as seen in selected cranial and ear surgery studies. Furthermore, patient-specific 3D-printed prosthetic molds have been used in chin augmentation surgery, resulting in both decreased surgical time and an improved esthetic outcome on account of the personal profile match. Finally, our research (Additional file 2) suggests that 3D-printing techniques can successfully be used to directly print the final implant, most commonly in cranial surgery. Cranial custom implants seem to be accurate and to decrease OR time, while being associated with improved clinical outcomes in nearly all the studies considered.

Likewise, 3D-printed trays and fixation plates improve medical outcomes and reducing operation room time for maxillofacial surgery. Moreover, one selected study presented the additional advantage of improved bone formation and angiogenesis with the use of custom implants.

Finally, complete dentures can also be made by rapid prototyping. The results vary, with one study mentioning lower esthetics for 3D-printed dentures and another study mentioning esthetics similar to standard dentures, while highlighting the advantages of face simulation before printing the final prosthetic.

### General

3D-printing techniques are widely used for medical purposes. In the majority of the studies selected here, the medical outcome is improved by the use of 3D-printing. However, we believe that the enthusiasm should be tempered somewhat, as only 14 % of the investigated studies supported this statement with numbers, making this major advantage rather subjective.

Operation time reduction is mentioned in nearly half of the selected studies and backed with numbers in only two-thirds of these cases. In general, most 3D-printing applications seem to reduce the OR time, but wide variances can be seen between the different usages. Some OR time reductions are too small to result in relevant benefits. Although OR time reduction is a major advantage that could contribute to significant financial reduction, the increased time needed for surgical planning is rarely considered. Few studies explicitly mentioned the increased preparation time or discussed whether outsourcing surgical planning is an option. According to two selected studies using surgical guides for knee arthroplasties, surgeons and patients spend more time preparing for surgery than can be reduced during the surgery. Furthermore, these studies suggest that planning might more accurate when performed by the surgeon than when outsourced.

Although the large majority of the selected studies do not mention exposure to ionizing radiation, two-thirds of the studies that do mention radiation report a decrease in this ionizing radiation. This can be explained by the high proportion of spinal surgery applications that mentioned decreased exposure to ionizing radiation, as fluoroscopic guidance is a well-known practice in that specific domain. It would be questionable to extrapolate this finding to other domains, as medical 3D printing requires CT scans or MRI. The first of these exposes the patient to a significant amount of ionizing radiation; fluoroscopic guidance, on the other hand, is not that frequently used.

Patients can additionally benefit from technology as anatomical models improve patient understanding of the pathology and procedure. This results in improved patient–doctor communication and greater patient satisfaction. Tactile anatomical models can also assist medical and surgical students to improve their knowledge.

Cost-effectiveness of the new technology is suggested in 7 % of the selected publications, but is nowhere supported by numbers. Other publications question the cost-effectiveness and conclude that the use of 3D printing is not cost effective. Several authors mention that the complexity of cases can justify the additional cost of surgical guides. The growing economic pressure on healthcare makes it increasingly important for researchers to consider the economic sides of new technologies and techniques. Even small analyses made by non economists can be an indication of whether a new technique tends to be cost-effective or not. Fuller cost-effectiveness studies would be needed to evaluate the acceptability of the technology, both for complex cases and for routine cases using 3D printing. Although this was one of the key points of this review, few data on it could be found in the literature.

The cost of 3D-printed parts depends heavily on the manufacturing facility. Cheap desktop 3D-printers allow cheap 3D models and guides, but have less quality approvals and controls than commercial manufacturers, who are required to meet high quality standards. Furthermore, the reported costs of self-printed parts differ from author to author, with few mentioning direct preparation costs (CT, MRI, multiple prints, software, and computer) or the time cost involved in designing the model. The heterogeneity of these printed parts prevents more in-depth analysis. Therefore, we would encourage future research to present the data in a much more transparent and objective way, and to make the first steps into cost-effectiveness calculations.

Although we considered additional articles found in the references of the selected publication, we are aware that some relevant articles might have been missed. We included case series and trials with four or more observations with the assumption that the most integrated practices will have publications stating their specific use. This means that subjects only reported in case reports could have been missed, even if they were well integrated. Surgical publications were considered and analyzed using an evidence table. Not all aspects that might be advantageous for a specific usage can be considered, especially when these advantages are not the direct result of the 3D-printed part. Medical 3D-printing applications used for testing, demonstrations, and training only were not incorporated in this review.

## Conclusion

3D printing is already well integrated in medical practice and the literature. Applications vary from anatomical models (mainly for surgical planning) to surgical guides and implants. The main advantages stated by the authors of the selected papers are reduced surgical time, improved medical outcome, and decreased radiation exposure. Unfortunately, the subjective character and lack of evidence supporting majority of these advantages does not allow for conclusive statements. The increased cost of this new technology, and the often limited or unproven advantages, make it questionable whether 3D printing is cost effective for all patients and applications. Several authors have indicated that medical 3D printing has greater advantages when used to handle complex cases and with less experienced surgeons.

## Additional files

**Additional file 1.** Data overview. List of included articles with extracted data for OR time, accuracy, radiation, ease of use, cost, cost effectiveness and outcome.

**Additional file 2.** Full evidence table. Extended version of Table 1 with subdivision per medical specialty.

## Abbreviations

AM: additive manufacturing
CAD: computer aided design
CAM: computer Aided manufacturing
CT: computed tomography
MR: magnetic resonance
OR: operation room
3D: three-dimensional
